# Phytoestrogens (Resveratrol and Equol) for Estrogen-Deficient Skin—Controversies/Misinformation versus Anti-Aging In Vitro and Clinical Evidence via Nutraceutical-Cosmetics

**DOI:** 10.3390/ijms222011218

**Published:** 2021-10-18

**Authors:** Edwin D. Lephart

**Affiliations:** Department of Cell Biology, Physiology and The Neuroscience Center, College of Life Sciences, Brigham Young University, Provo, UT 84602, USA; Edwin_Lephart@byu.edu; Tel.: +1-801-319-8173

**Keywords:** anti-aging, equol, estrogen, misinformation, nutraceutical, phytoestrogens, resveratrol, skin

## Abstract

The overarching theme for this review is perspective. Superfoods (a marketing term for fruits and vegetables, etc.) have a positive connotation, while many superfoods contain phytoestrogens, a term that is alarming to the public and has a negative connotation because phytoestrogens are endocrine-disruptors, even though they are strong antioxidants that have many health benefits. To understand phytoestrogens, this paper provides a brief summary of the characteristics of: (a) estrogens, (b) estrogen receptors (ER), (c) estrogen-deficient skin, (d) how perspective(s) get off track, (e) phytoestrogen food sources, and (f) misconceptions of phytoestrogens and food safety, in general, that influence person(s) away from what is true. Finally, a brief history of cosmetics to nutraceuticals is covered plus the characteristics of phytoestrogens, resveratrol and equol on: (g) estrogen receptor binding, (h) topical and oral dosing, and (i) in vitro, molecular mechanisms and select clinical evidence, where both phytoestrogens (resveratrol and equol) demonstrate promising applications to improve skin health is presented along with future directions of nutraceuticals. Perspective is paramount in understanding the controversies associated with superfoods, phytoestrogens, and endocrine-disruptors because they have both positive and negative connotations. Everyone is exposed to and consumes these molecules everyday regardless of age, gender, or geographic location around the world, and how we understand this is a matter of perspective.

## 1. Introduction

**Figure 1:**
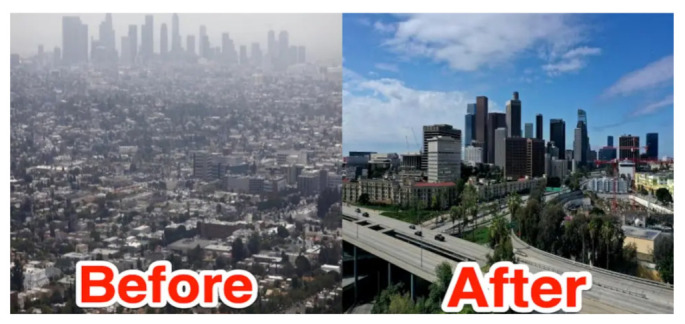
Before and after photos show how stay-at-home orders during COVID-19 helped Los Angeles significantly reduce its notorious smog [1]. Displaying clear-cut differences in smog levels.

**Figure 2:**
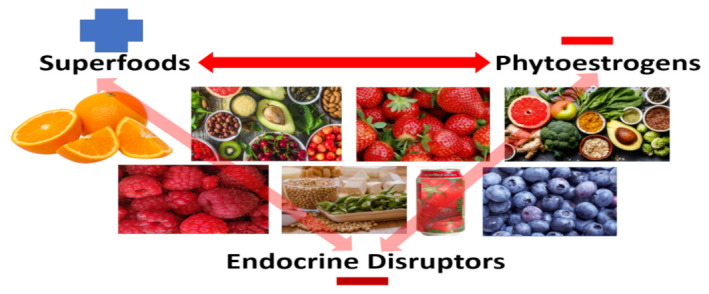
Superfoods have a positive connotation shown by the blue plus symbol because they contain antioxidants and other compounds that support good health, while superfoods also contain phytoestrogens, which have a negative connotation (red minus symbol) because they bind to estrogen receptors and hence are classified as endocrine-disruptors (deep-red minus symbol) that is alarming to the public. Thus, it is a matter of perspective of how each is perceived.

The theme for this review is perspective, covering all aspects of this review’s title. Viewing before and after photos show how COVID-19 stay-at-home orders helped Los Angeles significantly reduce its notorious smog [1]. The before and after photos are obvious, clear cut, and easy to understand because the perspectives are distinct (Figure 1). However, when superfoods (a marketing term) are compared to the term phytoestrogens, superfoods have a positive connotation even though they contain phytoestrogens [2,3]. Conversely, phytoestrogens are alarming to the general public and have a negative connotation because they have been classified as endocrine-disruptors, even though phytoestrogen are antioxidants that reduce inflammation and oxidative stress (by inhibiting oxidation produced by free radicals that damage cells/tissues) along with having other health benefits and are in superfoods [2]. This superfood concept not only applies to traditional land-plant sources, but marine-based compounds as well [4,5,6,7]. So, this understanding becomes a matter of perspective (Figure 2).

## 2. Characteristics of Estrogens

To understand phytoestrogens, one must first examine estrogen characteristics. All steroid hormones are derived from cholesterol (while phytoestrogens are not). For example, endogenous estrogen hormones such as 17β-estradiol are steroids with a cyclo-hexane parent chemical structure [8]. Notably, the most potent steroid hormone is 17β-estradiol (E2), meaning a low level (in picograms per milliliter) of this sex steroid hormone delivers a strong signal to cells/tissues throughout the body [9]. In fact, estrogen molecules are mole for mole, 100- to 1000-fold more biologically active or potent compared with their parent androgens [9].

While estrogens widely influence many important functions such as homeostatic actions, cell proliferation and death, liver protein expression, lipid metabolism, energy balance, glucose metabolism, immune and cardiovascular regulation, gonadotrophin feedback and gametogenesis, brain-neuronal development/memory processing and repair/neurodegeneration, bone growth, and others, this review is focused on estrogen and dermal health, especially in estrogen-deficient skin in women [9]. In women, E2 levels increase after puberty, peak in women during their late 20s, and begin to decline after age 30 from the ovaries and fall to zero after menopause [9].

### 2.1. Characteristics of Estrogen Receptors 

**Figure 3:**
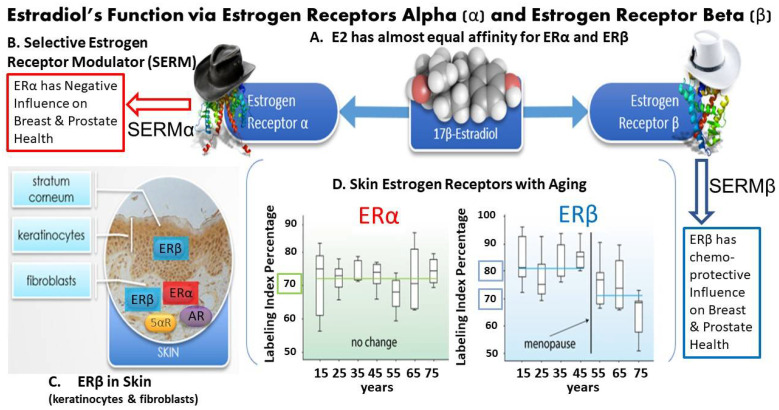
(**A**) The chemical filling model of 17β-estradiol shows almost equal affinity for estrogen receptor (ER) alpha (α) and ER beta (β) [8]; (**B**) Selective estrogen receptor modulators (SERMS) are compounds that have agonist properties to preferentially bind to ERα or ERβ, where SERMS binding ERβ have positive while SERMs binding ERα have negative impact on breast and prostate health; (**C**) displays the distribution of ERα (red), ERβ (blue), the 5α-reductase enzyme (5α-R in gold) that converts testosterone to the more potent androgen, 5α-dihydrotestosterone or 5α-DHT; and androgen receptor (AR in purple); (**D**) displays the labeling index (percentage) for ERα (red label with green shading) and ERβ (blue label with blue shading) with age in years.

E2 delivers its chemical message via estrogen receptor alpha (ERα) and estrogen receptor beta (ERβ) throughout the body including the skin [8,9]. The other classification of estrogen receptors (ERs), such as the G protein-coupled receptor (GPER) and mitochondrial receptor will not be covered herein since the GPER in skin has not been well studied and the ER mitochondrial receptor has not been identified in skin to date [9]. However, it should be noted that ERα and ERβ have almost equal affinity for 17β-estradiol [8]; see Figure 3A. ERα, activation is a major factor in reproductive cancers (e.g., breast and prostate), whereas ERβ activation appears to be chemoprotective at these tissue sites [9]; see Figure 3B. Finally, ERβ activation has been shown to promote wound healing independent of estrogen’s anti-inflammatory properties [9]. 

Selective estrogen receptor modulators (SERMs) at ERs have proven to provide skin benefits [9], as Wilkinson and Hardman demonstrated in their recent review on estrogen and SERMs in dermal health [10]. There is tissue-specific expression in humans of the ERs, where ERβ is more widely expressed in skin compared with ERα, and this is especially the case in the human scalp [8,10,11]; see Figure 3C. Inoue et al. examined skin ERs with aging in women and found that the labeling index for ERα remained stable from 15 to 75 years of age at approximately 72%, while the ERβ labeling index declined by approximately 15% from 82 to 70% after menopause [11]; see Figure 3D. 

Finally, it is known that the estrogen receptors are ancient and promiscuous, binding hundreds of compounds including estrogen analogs, estrogen metabolites, various SERMs, xenoestrogens such as phytoestrogens, marine algae including bacterial compounds and even include some androgen and progestogen compounds, which is uncommon compared to other nuclear steroid receptors [7,12,13,14,15,16,17].

### 2.2. The Endocrine System, Skin Health and Hormonal Imbalance Associated with Menopause

The skin is an endocrine organ where dermal cells contain the biochemical apparatus necessary for hormone production that influences local immune function [18]. Additionally, there are several hormones produced by the endocrine system from glands that secrete chemical messengers into in the bloodstream that bind to specific receptors in cells/tissues to maintain homeostasis in the body that also influence skin health [19,20,21,22]. Two of the most common regulatory messengers encountered in dermatology involve thyroid and cortisol hormones [19,20,21,22]. The thyroid hormone regulates the metabolic rate of the body, while cortisol also regulates metabolism, the immune response and helps the body respond to stress [19,20]. By binding to their receptors thyroid hormone helps regulate epidermal cell proliferation, differentiation, hair and nail growth, wound healing, and skin hydration by affecting the function of dermal fibroblasts [19,20]. Stress-induced cortisol levels can cause the skin’s sebaceous glands to produce more sebum (oil) and cause flareups in acne, psoriasis, eczema, hives, skin rashes and fever blisters [21,22]. Additionally, high cortisol levels can contribute to thinner sensitive skin (impaired barrier function), delayed wound healing, dark circles around the eyes, and increased fine lines and wrinkles due to the breakdown of collagen and elastin [21,22]. 

As covered in Section 2 above, the decline of estrogen levels with aging is also associated with reductions in circulating progesterone and androgen levels [23,24,25]. In fact, the progesterone production rate is approximately 29 mg/24 h in the mid-luteal stage of the menstrual cycle but drops to below 1 ng/mL in blood after menopause [9,26]. Additionally, it has been reported in women that androgens are essentially made from DHEA in peripheral tissue sites according to intracrinology [27], which also decline with aging and after menopause drops to low levels [23,28]. However, it is known that progesterone promotes skin elasticity [29] and has a higher affinity for the 5α-reductase type I enzyme (in dermal fibroblasts) compared to testosterone [30], which blocks testosterone from being converted to the more potent androgen, 5α-dihydrotestosterone (5α-DHT) that decrease dermal fibroblast viability and function [31,32]. Therefore, the small amount of circulating testosterone remaining after menopause may predispose to androgenic symptoms via its conversion to 5α-DHT with actions such as acne, increased facial hair growth and female pattern baldness [28]. Thus, the hormonal imbalance not only involves the lack of estrogen and progesterone after menopause, but the increased negative effects of androgenic hormonal actions in skin especially from 5α-DHT [32].

### 2.3. Characteristics of Estrogen-Deficient Skin

There are numerous reviews covering skin aging and estrogen-deficient skin [9,10,11,23,24,25,29], so in brief, estrogen-deficient skin (with aging and especially after menopause) displays increased dryness, wrinkles, perceived age, impaired wound healing, breakdown of collagen and elastin by matrix metalloproteinases (MMPs), decreased barrier function and antioxidant capacity, and a decline in defense against oxidative stress, hydration, etc. [9,10,11,23,24,25,29]. Furthermore, caution should be exercised in the administration of hormone replacement therapy (HRT) after menopause, due to the benefit/risk ratio [9,32].

## 3. How Do Things Get off Track? How Can Perspective Be Influenced Away from What Is True?

People’s viewpoints and perspectives vary greatly and are influenced especially by social media. For example, a Scientific American article published in December of 2020, “Information Overload Helps Fake News Spread, and Social Media Knows It,” demonstrated how modern technology has proliferated information to provide personalized recommendations that in turn amplified cognitive biases [33]. While individuals feel independent, they confuse popularity with quality information (confirmation bias) and tend to follow others who have similar viewpoints [33]. 

With this perspective in mind, here are three examples: (a) up to ten percent of Americans believe the moon landing (in July 1969) was fake, and younger-aged individuals are 6-fold more likely to express this viewpoint most likely because they did not watch the actual moon event [34], (b) when first introduced to the public in July of 1977, nuclear magnetic resonance imaging (MRI) during the cold war (1947–1991), patients were hesitant to undergo any sort of “nuclear” treatment because of the negative connotation (associated with destruction), so the “nuclear” term was dropped and the powerful imaging scan that uses radio waves and a powerful magnet linked to a computer is used by millions of people worldwide to “see” detailed images inside the body [35] and (c) in 1981, Roger Sperry won the Noble Prize in Physiology and Medicine for human split-brain studies conducted in the 1960s with his graduate student Michael Gazzaniga, who is now a noted neuroscientist and academic textbook author. The split-brain findings suggested that within the right-brain resided creative, artful, imagination and emotional components whereas the left-brain was more analytical, logical, scientific, and factually oriented where the media, popular and academic articles and books promoted the false ideas that individuals are dominant on one side of the brain or the other to explain personality and learning characteristics [36]. As Michael Gazzaniga described in his textbook, “Psychological Sciences,” “The hemispheres are specialized for certain functions, such as language and spatial relationships. However, a recent clinical study examined the brain activity in over 1000 individuals ages 7 to 29 that found no differences between people to the extent their right or left hemisphere was active” [36,37]. Thus, the concept of right-brain vs. left-brain types of people is a myth.

## 4. How Do We Know What Is True? Four Basic Elements

Traditionally, it encompasses four elements: (a) authority—of individuals, organizations or agencies that we trust or have faith in. However, what happens when a person with authority, a medical doctor, for example, spreads misinformation about COVID-19, resulting in controversy like this, “Now, thanks to the internet and social media, the misleading musings of a local doctor speaking before a school board can compete for attention with the recommendations of the CDC” [38]? (b) Repetition or tenacity—even when the information is unclear, confusing or incorrect. (c) A priori or reasonable—like the example shown in Figure 1, which displays before and after photos that show how COVID-19 stay at home orders helped Los Angeles significantly reduce its notorious smog. (d) Scientific analysis has always led the way throughout history; however, science, in many ways, is under attack even though the Latin root for science comes from the term “Scientia,” which means “knowledge or to know” [39]. In this regard, people, in general, prefer information from people they trust, who have like-minded perspectives and belong to their in-group where popularity outweighs qualified or data analysis and scientific reporting [33]. 

## 5. To Complicate Matters, “Natural,” “Pure,” “Clean,” “Organic,” and “Whole” When Applied to Food Sources May or May Not Be Entirely What One Perceives

To illustrate this perspective, three brief examples are poignant: (a) in 2015, the United States Food and Drug Administration (US FDA) published the Defect Levels Handbook: The Food Defect Action Levels of natural or unavoidable defects in foods that present no health hazards for humans [40]. In brief, this report outlines the acceptable levels of mold, maggots, insect larvae, insect fragments and rodent fecal material in various foods like berries (raspberries, etc.), cherries, corn, and wheat just to mention a few examples of defects in foods [40]. (b) In 2010, Dolan et al. reviewed the presence of toxins that occur naturally in foods [41]. The authors stated, “regardless of measures taken by regulators and food producers to protect consumers from natural food toxins (e.g., in fruits, wheat, and rye) consumption of small levels of these materials is unavoidable. Although the risk for toxicity due to consumption of food toxins is fairly low, there is always the possibility of toxicity due to contamination, overconsumption, allergy or an unpredictable idiosyncratic response” [41]. (c) In 2020, Palacios et al. reviewed the presence of naturally occurring hormones (protein, steroid hormones or phytoestrogens) in foods such as cereals, breads, chicken, eggs, fish, fruits, ham, legumes, meat/beef, milk, nuts/seeds, potatoes, soybeans, turkey, wheat, vegetables and yogurt, and they concluded, “hormone content typical serving sizes of consumed foods either- undetectable or fall well within safety guidelines without any adverse effects on health” [42]. Thus, while nature may be perceived as pure, it also represents a challenging environment for all living things on earth.

## 6. Phytochemicals, Polyphenols and Phytoestrogens

In 2014, Buckingham estimated that there are more than 20,000 natural products that have been identified [43]. Lui reported that thousands of individual dietary phytochemicals have been identified, for example, in fruits, vegetables, whole grains, legumes, and nuts, but a large percentage of them remain unknown [44]. 

**Figure 4:**
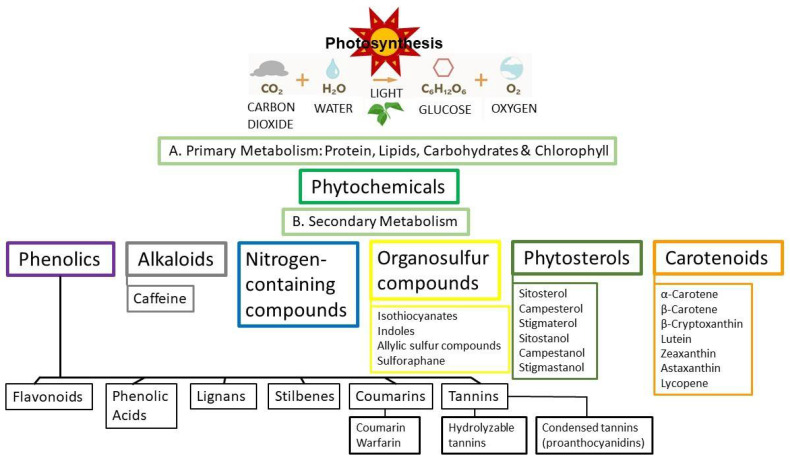
Photosynthesis—cartoon of life-giving oxygen generation plus (**A**) primary metabolism in plants leading to phytochemicals and (**B**) secondary metabolism leading to the major classes of compounds along with examples of phytochemicals under each classification and the major subgroups of phenols.

Life on earth is dependent upon photosynthesis by plants that involves the conversion of carbon dioxide and water in the presence of light from the sun to generate glucose and oxygen (Figure 4) [45]. Primary metabolism in plants includes the components protein, lipids, carbohydrates, and chlorophyll and leads to secondary metabolism that comprises phytochemicals (Figure 4) [45]. Phytochemicals can be divided into six major groups: (1) phenolics, (2) alkaloids, (3) nitrogen-containing compounds, (4) organosulfur compounds, (5) phytosterols and (6) carotenoids [44] (Figure 4). (While phytosterols are estrogenic and have a chemical structure similar to cholesterol, this phytochemical class will not be covered in this review.) Polyphenols are a subclass of phytochemicals that consist of six classifications: (1) flavonoids, (2) phenolic acids, (3) lignans, (4) stilbenes, (5) coumarins and (6) tannins (Figure 4) [44]. The focus of this review is on polyphenols, where more than 10,000 phenolic structures have been identified [46,47]. 

The lesser-known phenols are coumarins and tannins and will not be discussed (in depth) in this review. However, in brief, more than 1300 coumarins have been identified and are found in clover, lavender oil, woodruff, tonka beans, strawberries, cherries, celery, apricots, etc. [48]. Coumarins and warfarin act as anticoagulants that inhibit clot formation by competing with vitamin K are moderately toxic to the liver and kidneys and were banned as a food additive in the U.S. in 1954 [48]. Tannins are found in the bark of trees, wood, leaves, buds, stems, seeds/nuts, roots, thyme, fruits, pomegranates, strawberries, blueberries, tea, coffee, beer, wine, etc., and many researchers believe they have positive influences as anti-microbial agents, anthelminitic (to destroy parasitic worms), and protein bypassed effects in ruminants [49].

**Figure 5:**
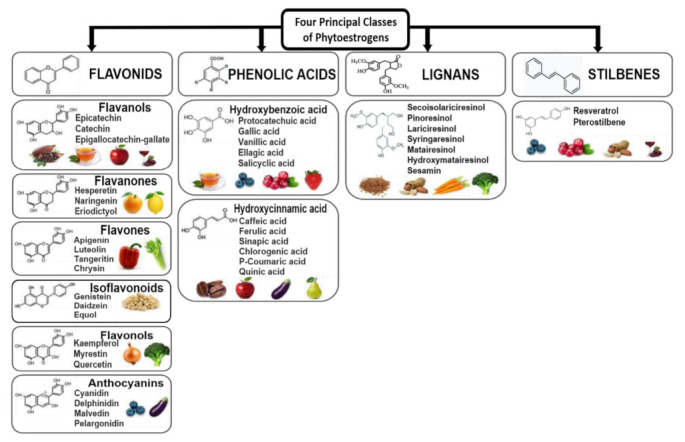
Four principal classes of phytoestrogens, the various subclasses, examples of chemical structures, chemical names and pictures of food products that contain these polyphenolic/phytoestrogens compounds [53] (use and modified with permission for BenthamScience ID # 600053254 and IN 16207).

Notably, the four principal classes of phytoestrogens, which are polyphenols, are (1) flavonoids, (2) phenolic acids, (3) lignans and (4) stilbenes (Figure 5). Recall that plants do not make estrogens, but contain compounds that are estrogen-like or estrogenic in their ability to bind the mammalian estrogen receptors (ERα and/or ERβ), and if the compounds have a higher affinity for ER subtypes, then they are classified as SERMs [9,10,12,13,16,17,23,29].

As displayed in Figure 5, the flavonoids have six subclasses that are (1) flavanols, (2) flavanones [and the flavanonols subset, the 3-hydroxy derivatives, e.g., (aromadendrin and taxifolin) found in grapes and red wine] [50], (3) flavones, (4) isoflavonoids, (5) flavonols and (6) anthocyanins, while the phenolic acids have two subclasses which are (1) hydroxybenzoic acid and (2) hydroxycinnamic acid (Figure 5) [45,47,51,52,53]. Remarkably, flavonoids account for approximately 60% of all polyphenols, while phenolic acids account for approximately 30% of all polyphenols [45,47]. Finally, there are several lignans and stilbenes, but the best-known stilbene is resveratrol (see Figure 5 for the four principal classes of phytoestrogens, the various subclasses, examples of structures, chemical names and pictures of food products that contain these polyphenolic/phytoestrogens compounds) [44,45,51,52,53,54].

All the possible food sources or food products for the four principal classes of phytoestrogen are not shown in Figure 5 and will not be given herein. However, a few examples will be described in brief for the flavonoids (since they represent 60% of dietary phytoestrogens) such as the isoflavonoids (daidzein, genistein and equol) and the flavonols (quercetin) that will be featured. Isoflavonoids are found in soybeans (soy food products, Asian foods, especially fermented foods), beans, peas, chickpeas, lentils, cabbage, lettuce, kale, peanuts, fruits, vegetables, onions, alfalfa, clover, spices, grains (wheat, barley, bran), cereals, seeds, Kudzu, cow’s milk and eggs [44,45,47,51,52], while the flavonol quercetin can be found in apples, cherries, berries, peppers, cruciferous vegetable (broccoli, cabbage and sprouts), spinach, kale, tea, herbs, tomatoes, citrus fruits, cocoa, cranberries, whole grains, asparagus, red wine, capers, onions, olive oil, and legumes [44,45,47,52,53].

For the phenolic acids, being the second most abundant subclass, accounting for 30% of dietary phytoestrogens, the hydroxybenzoic acids are found in tea, fruits, onions, and radishes [51,52], while the hydroxycinnamic acids are found in coffee, cinnamon, and fruits (such as apples, pears, cherries, plums, and blueberries), carrots, lettuce, eggplant, legumes, cereals, and grains (wheat) [52,55].

Lignans are found in flaxseed (flour, oil, etc.), linseeds, lentils, beans, peanuts, seeds/nuts, soybean, soy, chickpea, clover, rapeseed, sesame, whole-grain cereals such as wheat, oats, rye and barley, legumes, various vegetables (like carrots and broccoli), fruits (especially berries), dairy products, meat, fish, and seaweed [51,56,57,58].

Relatively low levels of stilbenes occur in the human diet; however, resveratrol is the best-known polyphenol/phytoestrogen that is in grape skins (especially red grapes), blueberries, raspberries, cranberries, mulberries, peanuts, and cocoa powder [35,36,41,46,59]. The pterostilbene compound is found in grapes, blueberries, mulberries, almonds, peanuts, and cocoa powder [53,60]. Particularly, pterostilbene is a dimethylated derivative of resveratrol, where the two methoxy groups increase the lipophilic properties and oral absorption to approximately 80% bioavailability compared to 20% for resveratrol [53,60].

Although not the focus of this review, the phytoestrogens mentioned in this section have many attributes, including antioxidant, anti-inflammatory, anti-bacterial, anti-fungal, anti-viral, anti-hypertensive, anti-convulsant and have demonstrated promising effects in the prevention of various ailments, such as heart disease, cancer, stroke, diabetes, obesity, osteoporosis, many neurodegenerative disorders such as Alzheimer’s disease [8,9,10,44,45,47,48,49,50,51,52,53,54,55,56,57,58,59,60,61] and finally age-related decline in human skin health [8,9,10], which will be covered in detail in the latter sections.

Finally, as demonstrated by the list of food sources and food products above, it is impossible to exclude phytoestrogens from human consumption. For example, “humans are exposed to these phytoestrogens compounds from different plants and food sources regardless of age, gender or geographical location with scientific data to support a consumption/exposure record that appears to be safe” [8,62,63]. 

### 6.1. Soy/Phytoestrogen Controversies

In this review, the terms soy, isoflavones and phytoestrogens can be used collectively, since soybeans are a high source of isoflavonoids, and in turn, flavonoids are phytoestrogens. Thus, these terms can be used interchangeably [44,45,47,52,53]. Conversely, from a scientific perspective isoflavones should not be equated with estrogen and soy foods should not be equated with isoflavones [64].

The reasons the soybean and other phytoestrogens containing foods have gone through cycles of critical scrutiny are unknown and that resulted in safety controversies [65]. The soybean is a vegetable protein with no cholesterol or lactose and is a good source of fiber and complex carbohydrates [65]. Like the controversies associated with resveratrol, the phytoestrogen controversies may be due to factors of disparate doses and variable experimental designs especially from in vitro and animal studies not supported by human study results, especially in the health of postmenopausal women [59,66]. A recent review that examined soy foods, isoflavones, and the health of postmenopausal women concluded that “the clinical and epidemiologic data indicate that adding soy foods (or phytoestrogens) to the diet can contribute to the health of postmenopausal women” [66]. Food safety has been addressed recently in vegetarian diets [67], where soy, soy foods and phytoestrogens were examined along with concerns about infant nutrition/development, pro-cancer disorders, thyroid disturbances, sex hormone perturbations and altered reproductive functions [65,67]. The health effects of soy/phytoestrogens have been investigated for more than 30 years, with more than 2000 peer-reviewed articles published annually on this topic, including reviews on each health controversy mentioned above [9,44,45,47,52,53,54,59,60,61,62,63,64,65,66,67,68,69,70]. However, in brief, each of the four major health controversies is addressed below. 

#### 6.1.1. Soy/Phytoestrogens Increase the Risk of Cancer

No. Scientific evidence is accumulating to suggest that soy/phytoestrogens may have a role in preventing chronic disease, and breast and prostate cancers [52,59,61,64,68,71,72,73,74,75,76,77]. The strategic director (Dr. McCullough) of nutritional epidemiology for the American Cancer Society (USA) recently stated, “So far, the evidence does not point to any dangers form eating soy (*phytoestrogens*) in people, and the health benefits appear to outweigh any potential risk. In fact, there is growing evidence that eating traditional soy foods may lower the risk of breast cancer” [78].

#### 6.1.2. Soy/Phytoestrogens Negatively Impact Thyroid Function

No. Several clinical studies have suggested the soy/isoflavone/phytoestrogens do not have any effects on the thyroid or thyroid function [64,79,80,81,82]. Additionally, a report from the European Food Safety Authority [83], along with a randomized, double-blind, crossover clinical study showed no alterations in thyroid function tests in patients with subclinical hypothyroidism [84] and a meta-analysis reported phytoestrogens have no effect on thyroid hormones [85]. Moreover, Dr. Nippoldt in 2019 from the Mayo Clinic recently stated, “There’s no evidence that people who have hypothyroidism should avoid soy (*phytoestrogens*)…” [86].

#### 6.1.3. Soy/Phytoestrogens Have Feminizing Effects on Males (Neonates, Infants, or Adults)

No. There were no differences reported for the following parameters compared to controls for: (a) brain wave activity [electroencephalographic activity (EEG patterns at 3, 6, 9, or 12 months of age [87]; (b) ultrasonographic patterns of reproductive organs in infants or at 5 years of age [88,89]; and (c) reproductive hormone levels in males (young or adult) [62,64,68,69,70].

#### 6.1.4. Soy/Phytoestrogens Alter Reproductive Function in Males or Females (Puberty or Biomarkers in Adults)

No. There had been no indications that soy phytoestrogens cause: (a) alterations in hormone levels [62,64,68,69,70,90,91], (b) gynecomastia (breast enlargement) [64,68,69,70,92], (noteworthy: in 1979 in The Lancet [93] and in 1985 in the Journal of Pediatrics [94] it was reported that estrogens in meat consumed by boys and girls were associated with breast enlargement [93,94]), (c) erectile dysfunction [64,65,66,67,68,69,70], or d) decrease in sperm count [64,68,69,70].

#### 6.1.5. Summary and Conclusions on Controversies Associated with Phytoestrogens

In connection to the above topics, in early 2000, Dr. Stephen Safe published a review, “Endocrine Disruptor and Human Health—Is There a Problem? An Update”, where he covered the hypothesized environmental exposure to estrogenic chemical and the related endocrine-active compounds that might be responsible for a global increase in breast cancer and decrease male reproductive capacity, including declining sperm counts [95]. Additionally, in this review, endocrine-disruptors in the diet were covered. The summary of this review stated, “the role of endocrine disruptors and human disease has not been fully resolved however, at present the evidence is not compelling” [95].

Finally, an update to this perspective was given in 2017 by Schwarcz from the Office of Science and Society at McGill University that stated, “So, what’s the point? We are awash in dietary estrogens, yet virtually every day brings some alarmist news about some synthetic chemical found in plastics, cleaning agents or cosmetics that is supposedly harming our health because of its estrogenic effect. This in spite of the fact that these are found in smaller amounts and have much weaker estrogenic potential that compounds found in plants, meat and dairy products. I’m not suggesting there is no issue here… But, there is an undue emphasis on synthetic chemicals that may have estrogenic activity. I’m quite sure I’m getting more estrogenic compounds from my humus (chickpeas) than from the plastic container in comes in” [96]. Therefore, perspective is paramount in understanding the controversies associated with superfoods, phytoestrogens, and endocrine-disruptor compounds because they have both positive and negative connotations. Everyone is exposed to and consumes these molecules everyday regardless of age, gender, or geographic location around the world [8,59,62,63,64,69,96], and how we understand this is a matter of perspective.

## 7. Polyphenols/Phytoestrogens as Nutraceutical-Cosmetics for Skin Health

### 7.1. Short History: From Ancient Cosmetics to Nutraceutical-Cosmetics Today

The history of cosmetics comes first from ancient Egypt in approximately 10,000 Before Christ (BC) or Before Common Era (BCE), where women and men used scented oils and ointments for hygienic purposes, to moisten their skin plus cover up body odor and charcoal or soot were used as eyeliner to ward off evil spirits [97,98,99,100]. In ancient Greece, powdered chalk and white lead was used as face paint, where porcelain skin indicated wealth and beauty [98,99]. In ancient Rome, spoons and mirrors were used to see wrinkles and sun or age spots, where treatments included creams and ashes [98,99]. Approximately 1000 AD or CE medical physicians used perfume for deodorant and Japanese Geishas used rice powder and bird droppings to lighten their skin [98,99]. During the Middle Ages, skin lightening continued to be associated with wealth and health; however, those using cosmetics were negatively perceived as “painted individuals” with lower moral standards [98,99]. Western royal society from the Elizabethan era to the late 19th century and on into the 20th century greatly impacted the transition of cosmetics from flamboyance to improvement in pleasant odors, physical appearance, and attractiveness, where science and technology offered “cutting-edge” skin care products not to only the wealthy, but also to mass markets to improve everyone’s outward look along with increased awareness of safety in cosmetics [97,98,99,100]. During the roaring twenties, beauty, glamour, and prestige were highlighted until the great depression and with the events of the second world war, when movie stars made cosmetics an aspiration for all women [98,99,100]. Today, the internet era brings instant information and anti-aging product delivery from companies to customers looking for quick results from personal care products using “natural” and personalized treatments with multifunctional active ingredients.

**Figure 6:**
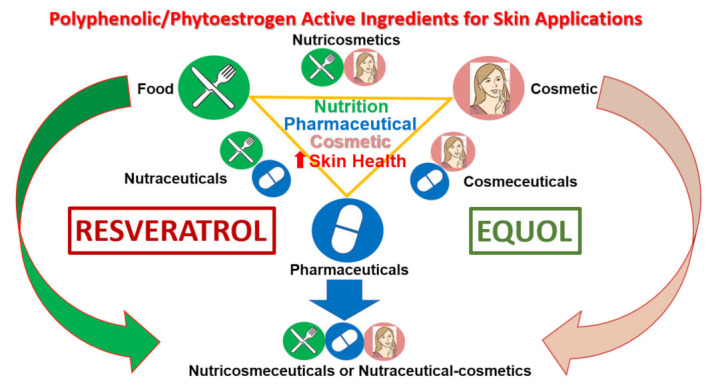
Resveratrol and equol as polyphenolic/phytoestrogens as active ingredients for topical and oral skin applications [101] (use and modified with permission-Elsevier ID 1146147).

The concepts in cosmetics and dermatology have evolved dramatically since the introduction in 1960s, when the terms “cosmeceutical” was made and later in the 1980s “nutraceuticals” started to gain recognition, which has been reviewed in detail by Faria-Silva et al. (2020) [101]. However, in brief, the term cosmeceutical was first introduced by Raymond Reed in 1962 [101] and was transformed by Albert Kligman in the 1980s based upon tretinoin’s mode of action in treating UV-damaged skin [102]. It is interesting to note that skin care and cosmeceutical recommendations are often discussed in dermatology visits based upon a cross-sectional survey of dermatology residents [102]. Presently, the nutraceutical concept has expanded, from compounds found in foods (such as fruits and vegetables with high antioxidant levels) to benefit health, in general, to the consumption of foods or oral supplements that produce enhancements in skin health [101]. This is a growing market that highlights the concept of ‘beauty from the inside” or “feeding the skin” offering compounds to improve dermal well-being [101]. The connection between nutrition and proper skin health has been validated by the role vitamins, minerals and polyphenols play in protecting, maintaining, and enhancing skin components and biomarkers to function at optimal levels to produce anti-aging effects [98,99,100,101,102]. Food-derived bioactive compounds with anti-aging potential for nutricosmetic and cosmeceutical products has been reviewed recently by Hernandez et al. in 2020 [103]. In this regard, the phytoestrogens, resveratrol and equol will be highlighted in the following sections as active ingredients from food sources as topical or oral skin care treatments with pharmaceutical-like actions from a nutricosmeceutical and/or nutraceutical-cosmetic perspective (see Figure 6). 

### 7.2. Plants and Cosmetic Innovations

Plants have been used in cosmetic products since ancient times (see above) and produce a wide variety of metabolites responding to the environment they live in [97,104]. This is not only true for cosmetics, but natural products/compounds represent up to 40% of pharmaceuticals and therapeutic agents according to the USFDA [105]. Plants have played a vital role by providing ingredients to protect the skin, enhance dermal health and appearance to support and feed feelings/thoughts of attractiveness and well-being [97]. The connection between estrogen levels and dermal aging in women has been reviewed in detail, where a positive correlation between circulating estrogens and: (a) perceived age, (b) attractiveness, (c) enhanced skin health, and (d) facial coloration were observed [32]. The loss of estrogen, especially at/after menopause represents dramatic changes in skin components, attractiveness, and general health [9,10,23,24,25,29,32,99,100,106,107]. Modern cosmetics not only improve hydration, but improve the skin barrier, supporting structural elements like collagen and elastin and also protect against indoor and environmental pollutants [104,108]. The worth of the global cosmetic market is forecasted to reach 542 billion USD and the US cosmetic market 70 billion USD by 2022 covering skin and sun care, hair, deodorants, makeup, color cosmetic and fragrance products distributed by retail stores, brand outlets, direct and internet sales [104]. The major classes of cosmeceuticals include sunscreens, retinoids, antioxidants [vitamin C, vitamin E, vitamin B3 (niacinamide or nicotinamide), and coenzyme Q10], exfoliators, and pigment-lightening agents [108].

## 8. Brief Historical Background of Resveratrol and Equol

### 8.1. Scientific Literature Comparison of Resveratrol and Equol

Resveratrol is in at least 72 different plant species and is found in two isoforms: *trans*-resveratrol and *cis*-resveratrol [109,110]. The trans-resveratrol isomer, due to the 4′-hydroxyl group, has greater biological activity and was first discovered in 1939 in the roots of white hellebore (*Veratrum grandiflorm*) [111]. Due to the high abundance of resveratrol in Japanese knotweed, it is extracted from this source to produce high-purity material for commercial products [112,113].

Resveratrol is a pleiotropic polyphenolic/phytoestrogen belonging to the stilbene family and is one of the most investigated bioactive compounds found in foods (since the discovery reported in Science by John Pezzuto’s laboratory in early 1997 demonstrating its anti-cancer activity) [59,109]. Interestingly, after this report, the sale of grape products containing resveratrol, particularly red wine, significantly increased [59]. From 1997 to 2018, a total of 20,459 journal reports have been published on resveratrol covering basic and clinical research especially pertaining to health benefits [59]. Today, each year, over 2000 journal reports are published on resveratrol [59]. 

Equol has a chiral center at carbon 3, and thus can exist in two mirror image forms or enantiomers (S-equol and R-equol) [8,73,109,114]. Initially, it was thought that S-equol was the exclusive enantiomeric form produced by human intestinal metabolism (from daidzein, its precursor compound) [115], but as early as 1986, equol was found in cow’s milk [116] and since then, S-equol and R-equol have been found in plants, food, and animal products [8,117]. Commercially, high-purity racemic equol (a 50 percent mixture of the S- and R-isomers of equol) are biosynthesized from high-grade daidzein for consumer products. While not as dramatic as resveratrol, the polyphenolic/phytoestrogen, equol, is a relatively new compound for cosmetic or topical use [8]. In the late 1990s, when the “equol hypothesis” was proposed, which implied health benefits in humans (protection against breast and prostate cancer), there was increased research attention on this isoflavonoid compound [114]. In this regard, the concept of equol producers vs. equol non-producers (via human intestinal flora metabolism) was introduced and reviewed elsewhere [8,114]. From 1980 to 2000, approximately one hundred cumulative reports were published; however, to date, over 2000 journal articles on equol have been reported [109,114]. Both resveratrol and equol have applications to improve health and prevent/treat disease [45,47,52,53,54,58,59,61,64,66,71,72,73,74,75,76,77,109,110,113,114,118]; however, this review is focused on skin applications in estrogen-deficient skin. 

### 8.2. Comparisons of 17β-Estradiol, Resveratrol and Equol

**Figure 7:**
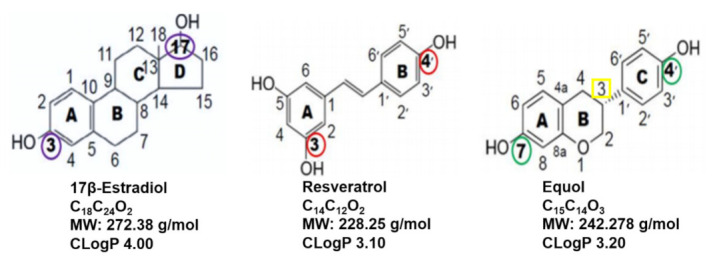
Comparison of the chemical structures, molecular formulas, molecular weights and CLogP values among 17β-estradiol (E2), resveratrol and equol are shown. The CLogP = the logP value of a compound is its partition coefficient, which represents lipophilicity. In this figure, all-trans resveratrol and equol (with a chiral carbon at position 3—yellow rectangle) represents R-equol, racemic equol or S-equol (redrawn with permission from MDPI Journals [109]). For all three compounds, the functional hydroxyl (OH groups), which enable binding to ERs and are indicated by color circles: purple at carbon 3 and 17 for 17β-estradiol; red at carbon 3 and 4′ for resveratrol; and green at carbon 7 and 4′ for equol.

The classification of resveratrol and equol as phytoestrogens is due to their: (a) similar chemical structures to E2, (b) the ability to bind ERs and (c) comparable molecular weights to E2 and (d) similar lipophilic characteristics [109]. The chemical structures, formulas, molecular weights, and lipophilic parameters of E2, resveratrol and equol are displayed in Figure 7. Recall, E2 is derived from cholesterol, while resveratrol and equol are secondary metabolites found in plants and food products [8,53,59,73,109,112].

### 8.3. ER Binding Characteristics of Resveratrol, Equol and Topical/Oral Dosing

Estrogen receptor (α and β) binding characteristics of resveratrol and racemic equol (including R-equol and S-equol) compared to 17β-estradiol (E2) have been reviewed elsewhere [109]. However, in summary, resveratrol is classified as a weak mixed agonist/antagonist for ER α and β [109,119]. For example, the binding of both ERs is very weak or more than 1000-fold less potent compared to E2, thus, resveratrol differs from other phytoestrogens that bind ERβ with higher affinity than ERα [109,119,120]. Conversely, racemic equol binds ERβ 35-fold lower than E2, which is 10-fold higher than its affinity for ERα [109]. Since equol has a chiral carbon and can exist as isomers, the binding characteristics of R-equol and S-equol have been examined for their affinities for ERs. S-equol binds ERβ with approximately 20% affinity (1/5- or 5-fold less) than E2, while having low affinity for ERα [109]. Thus, S-equol is classified as a SERM with a high affinity for ERβ [8,109,114]. On the contrary, R-equol has a weak affinity for either ER (more than 200-fold lower for α and more than 100-fold lower for β than E2) and thus, in general, has weak estrogenic properties [109].

Topical dosing based upon in vitro skin data (stimulation of skin biomarkers, human skin penetration and dermal metabolism studies) and clinical investigations, resveratrol (and its analogs) ranges from 1 to 3 percent in most personal care products [9,97,108,109,121,122]. However, microencapsulation or nanoparticle technology greatly improves delivery and hence decreases the concentration of resveratrol needed in topical formulations [123]. Human skin penetration profiles for resveratrol display a rapid increase by 6–7 h followed by a rapid decline into the epidermis and onto the dermal layers [98,110,123]. For equol, due to its unique potent epidermal actions and long-acting skin penetrating profile up to 28 h after a single dose, the concentration in personal care products ranges from 0.1 to 0.7 percent depending on the cosmetic application [8,9]. On this point, in preliminary studies, collagen was stimulated at 10 µM for resveratrol vs. 10 nM for equol in short-term human monolayer dermal fibroblast cultures [8,109,121].

The pharmacokinetics of resveratrol and equol are very different in many respects. Oral dosing for resveratrol presents a challenging problem due it is low bioavailability [124,125]. Following oral administration in humans, 75% of resveratrol is absorbed possibly by transepithelial diffusion. However, oral bioavailability is low (<1%) due to rapid and extensive metabolism in the intestine and liver (first-pass effect, conjugation), resulting in exceptionally low circulating levels of active compound, which require large oral dosing between 0.5 and over 1 gram of supplementation per day to furnish plasma levels sufficient to be efficacious [126,127]. On the other hand, equol is not conjugated to the same extent as resveratrol, oral dosing for equol is dramatically lower, where 5–10 milligrams per day is effective in improving skin health [117]. Pharmacokinetic studies examining oral equol administration displayed similar profiles for r-equol and s-equol, where there was a rapid rise after 2–2.5 h of ingestion with half-life intervals for both isomers at 7 to 8 h [128]. Oral resveratrol administration displayed a similar pharmacokinetic profile to that of equol with a half-life of 8 to 9 h [124,125,126,127], but with much lower bioavailability.

### 8.4. In Vitro/In Situ Evidence of Resveratrol and Equol for Nutraceutical-Cosmetics

#### 8.4.1. Human Gene Expression of Skin Biomarkers

Our laboratory along with our research associates have conducted several human skin gene expression studies on resveratrol (plus various resveratrol analogs) and equol (including racemic equol and the R- and S-isomers) using microarray analysis [31,109,121,122,129,130,131]. Notably, the addition of an acetoxy group on carbon 4′ of the resveratrol parent molecule generated an ester that increased the lipophilic and biological properties in skin studies [121]. The human skin data among resveratrol, the analog 4′ acetoxy-resveratrol, R-equol, racemic equol and S-equol for five major classifications of human skin gene biomarkers: (1) anti-aging and aging factors, (2) extracellular matrix proteins, (3) collagen and elastin degrading enzymes (matrix metalloproteinases), (4) antioxidant and heavy metal binder that are anti-inflammatory mediators and (5) various inflammatory factors (interleukins, TNF and COX) are summarized in Table 1.

In general, all-trans resveratrol was effective along with 4′ acetoxy-resveratrol, but racemic equol displayed the most efficacious values for the various human skin biomarkers followed by R-equol, while S-equol did not show any significant alterations in the collect data. The 4′ acetoxy-resveratrol analog and racemic equol compounds were further analyzed in preparation of clinic testing, covered in Section 8.4.3.

#### 8.4.2. Human Skin Cell Culture Studies and Molecular Mechanism of Action

Several studies have been performed on both all-trans resveratrol and (racemic) equol, but the most novel aspects of each phytoestrogen will be summarized in this section emphasizing their protection against oxidative stress, since this plays a critical role in human skin aging and dermal damage [8,9,109,110,113,126]. It should be noted that both resveratrol and equol act as strong antioxidants with higher antioxidant activity than vitamin E or vitamin C [8,109,110,113,126,131]. 

**Figure 8:**
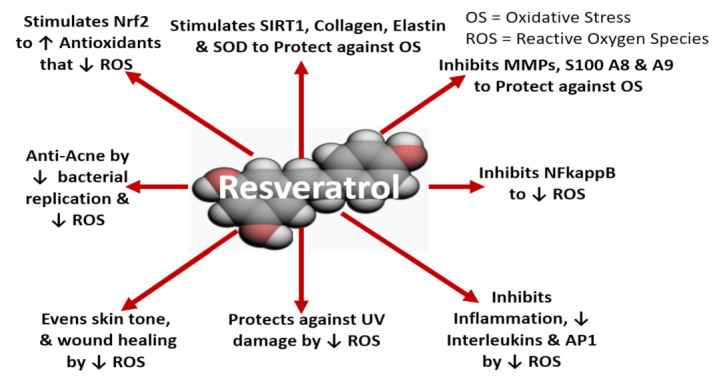
In vitro: how resveratrol works as an anti-aging skin molecule. Symbols/Abbreviations: SIRT = sirtuin activator 1 or NAD-dependent protein deacetylase sirtuin-1, an anti-aging factor; SOD = duperoxide dismutase (1 and 2), an antioxidant enzyme that protects against oxidative stress; MMP = matrix metalloproteinases (1, 3 and 9), an enzyme that breakdown collagen/elastin fibers; S100 A8 and A9 proteins = calcium-binding proteins (A8 and A9), related to skin aging, skin inflammation and photoaging; NFkappB = nuclear factor-kB, a transcription factor essential for inflammatory responses (infection/chronic inflammation, etc.); AP-1 = activator protein 1, a transcription factor that regulates gene expression of cytokines, stress, infections, etc.; Nrf2 = nuclear factor erythroid 2-related factor, a transcription factor that regulates production of antioxidants and detoxifying enzymes to combat oxidative stress.

How resveratrol works as an anti-aging skin molecule is shown in Figure 8. In several studies, resveratrol has been shown to: (a) stimulate nuclear factor erythroid 2-related factor (Nrf2; a master biofactor that increases antioxidant and detoxifying enzyme production) and increase SIRT 1 and 2 (anti-aging factors), collagen, elastin (extracellular matrix proteins) and superoxidase dismutase (SOD; an antioxidant enzyme) [109,113,126,131], (b) inhibit matrix metalloproteinases (MMPs enzyme that breakdown collagen and elastin) and the calcium-binding proteins S100 A8 and A9 (that are skin aging, inflammation and photoaging biomarkers) [109,126,132,133,134]; along with blocking the actions of NFkappB (nuclear factor-kB, a transcription factor essential for inflammatory responses (infection/chronic inflammation, etc.), interleukins and AP1 (activator protein 1, which regulates gene expression in response to oxidative stress/inflammation) [113,126,131,132,133,134]. Additionally, resveratrol protects against UV skin damage [110,113,126,132,133,134], has been shown to increase wound healing and skin lightening plus acts as an anti-acne agent by decreasing bacterial replication as well as inhibiting the inflammatory response [126,133]; see Figure 8.

**Figure 9:**
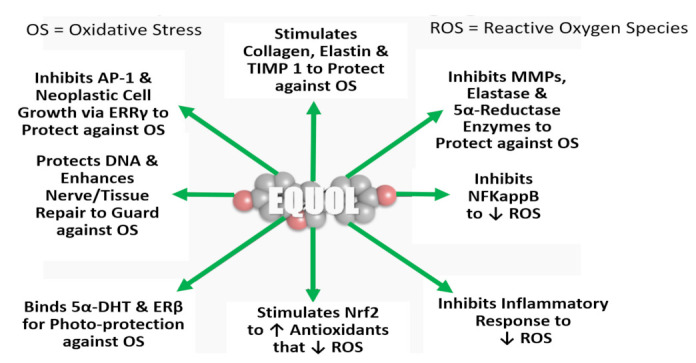
In vitro: how equol works as an anti-aging skin molecule. Symbols/Abbreviations: see Figure 8 legend, plus, TIMP 1 = Tissue Inhibitor of Matrix Metalloproteinase 1, inhibits the action of MMP 1; Elastase = enzyme that breaks down elastin; 5α-reductase enzyme = the steroid enzyme that converts testosterone to the more potent androgen, 5α-dihydrotestosterone (5α-DHT) that inhibits dermal health 5α-DHT = 5α-dihydrotestosterone, the more potent androgen that has negative impacts on skin health [8,32,33]; ERβ = estrogen receptor beta, that is abundant in human keratinocytes and fibroblast which phytoestrogens can bind; estrogen-related receptor (ERR) gamma (γ).

How equol works as an anti-aging skin molecule is shown in Figure 9. From multiple investigations, equol (mostly racemic, but some that studied S-equol) showed: (a) stimulation of collagen, elastin and TIMP 1 (Tissue Inhibitor of Matrix Metalloproteinase 1; inhibits the action of MMP 1) [8,109,118,129,135] (remarkably, in one study racemic equol stimulated SIRT 1 gene expression by almost 2-fold over controls [129], (b) inhibited MMPs (1,3 and 9), elastase and the type 1 5α-reductase enzymes [8,109] plus NFkappB and the interleukin inflammatory biomarkers (IL-6, IL-8 and COX-1) in human skin cells [8,109,118,136], (c) inhibition of AP-1 and neoplastic growth via estrogen-related receptor (ERR) gamma (γ) [8,137,138], (d) like resveratrol, equol stimulated Nrf2 [8,109,139], and (e) both R-equol, racemic equol and S-equol binds the potent androgen, 5α-DHT (5α-dihydrotestosterone, the more potent androgen that has negative impacts on skin health) and ERβ for photoprotection [8,31,32,109,140,141]. Particularly, equol’s photoprotection appears to be partially due to its antioxidant capacity and is dependent on stimulating the expression of metallothionein [142,143]. Finally, equol has been shown to protect DNA from oxidative stress and enhanced nerve/tissue repair [8,31,109,140,141]; see Figure 9.

Notably, both resveratrol [9,132,133,134,140] and equol [9,144,145] have been reported to improve skin health in clinical settings, which is covered in the next section.

#### 8.4.3. Clinical Skin Studies Examining Resveratrol and Equol: Oral and Topical

Not surprisingly, there have been more resveratrol clinical studies reported compared to equol and several reviews have summarized resveratrol’s positive enhancement on human skin health [110,113,126,133,134]. Although in recent years equol has gain attention in topical and oral dosing to improve skin health [9,105,129,130].

The topic of oral dosing to improve skin health (from the inside out) has been reviewed elsewhere [146]. In this review, Woodby et al. concluded that “dietary intervention alone is inadequate to prevent/treat skin conditions, primarily due to skin biology… the outermost layers of the epidermis are removed from the blood supply and ensuing nutrient delivery… Thus, …a two-pronged approach, utilizing both topical and oral intervention, is needed…” [146]. Thus, most skin care professionals would agree with this conclusion, although the percent contribution of each component (outside vs. inside may be debated). 

Therefore, a restricted number of oral and topical studies will be presented to demonstrate the best direct effectiveness of either phytoestrogen treatments: resveratrol or equol. Even though the bioactivities of phytoestrogens and resveratrol have been reviewed [147,148], the challenge of resveratrol research for estrogen-deficient skin during menopause for both topical and oral studies is limited, especially finding only this active ingredient (resveratrol) in the formulation without the addition of other skin-enhancing compounds. 

With that caveat for oral dosing with resveratrol, one prospective study examined twenty-nine women with visible signs of facial skin aging (36 to 76 years of age) that took two oral supplements daily for a total dose of 100 mg resveratrol plus 1000 mg of collagen [149]. After six months, the subjects had significant improvements in facial pores, ultraviolet spots, wrinkles, and skin tone with no adverse events reported [149]. The author’s admission included not knowing which active ingredient (collagen, resveratrol, or both) resulted in the positive improvement of the skin parameters. 

S-Equol was examined in postmenopausal women in a pilot randomized placebo-controlled trial for 12 weeks in 101 Japanese women who were equol non-producers [144]; 34 subjects in the placebo group, 34 subjects in the 10 mg dose and 33 subjects in the 30 mg dose of equol per day [144]. The skin parameters measured included crow’s feet wrinkles around the eyes, hydration, trans-epidermal water loss and elasticity, which significantly improved for both equol dosing treatments (i.e., 10 mg and 30 mg per day) compared to the placebo-control group values and with no adverse events reported [144]. These data suggest that S-equol oral supplementation may have beneficial effects on skin health in postmenopausal women with estrogen-deficient skin [144]. 

In connection with oral dosing of resveratrol and equol a report in 2017 by Davinelli et al. studied 60 menopausal women (50–55 years of age) in a randomized, placebo-controlled investigation on the influence of supplementation with equol (10 mg) and resveratrol (25 mg) per day for 12 weeks on hot flashes, anxiety, and depression symptoms [150]. The author concluded the 12-week dietary supplementation with a combination of equol and resveratrol significantly improved the menopause-related quality of life parameters in healthy postmenopausal women [150].

Finally, a recent placebo-controlled pilot study in men (37 to 56 years of age) showed that oral supplementation with 6 mg of racemic equol per day for 12 weeks significantly improved skin health parameters such as wrinkles, smoothness, skin tone (discoloration) and hydration [117].

For topical dosing with resveratrol, Farris et al. in 2014, reported the influence of 1% resveratrol, 0.5% baicalin (a flavonoid glycoside with anti-inflammatory and other skin benefits) and 1% vitamin E in 55 women (40–60 years of age) for 12 weeks that showed significant improvement in lines and wrinkles, firmness, elasticity, pigmentation, radiance, and smoothness [151]. Additionally, in a subset of 10 women, 2 mm punch biopsies were taken and the skin biomarkers COL1A1, COL3A1, PRKAA1, SOD, VEGFA, and HO-1 displayed significant increases in gene expression reflecting the facial improvements seen in the other quantified skin parameters [151]. Again, the limitations of this topical study are knowing what active ingredients contributed towards the reported improvements in skin health.

In another topical study that specifically examined a 2% emulsion of all trans-resveratrol, Brinke et al., in 2021, reported that applying the treatment twice daily for 8 weeks in 20 women (30–35 years of age) resulted in significant improvements in the quantified parameters of skin elasticity, barrier function, smoothness, thickness and density without adverse events [152]. From this study, the skin improvements can be directly related to the resveratrol treatment and not to other active ingredients in premenopausal women.

The final topically applied resveratrol treatment involved testing an analog of resveratrol, 4′ acetoxy-resveratrol (4AR) examined in a randomized, single center 12-week study of 36 women (ages 34–64 years old, where 56% of the women were postmenopausal for at least 2 years) with mild to moderate skin aging [9]. A 1% 4AR cream applied to the face/neck region twice per day for 12 weeks significantly enhanced the skin parameters of skin firmness, smoothness, even tone, wrinkles, radiance, pore size, spots/discoloration and hydration that ranged from 63% to 83% improvement over baseline [9], suggesting this resveratrol analog may be beneficial in estrogen-deficient skin.

For equol topical applications, two investigations reported promising results. In the first study, Magnet et al., in 2017, reported that topical equol administration (applied twice per day) for 8 weeks in 64 women (40–60 years of age) improved the structural and molecular skin parameters: roughness, texture, smoothness, firmness, elasticity and significantly decreased methylation and teleomere length in skin cells [145]. Additionally, the women did not show a significant difference in topically applied equol versus micro-encapsulated equol, suggesting the delivery was not enhanced by microencapsulation [145], which may relate to equol’s unique epidermal delivery mechanism over time [8,153].

In a second study, Lephart and Naftolin, in 2021, reported from a randomized, 12 week single-center study of 59 women (ages 40–70 years old), where 76% of the women were postmenopausal for at least 3 years) with mild to moderate skin photo-aging [9]. A 0.3% equol lotion applied to the face/neck twice daily after 12 weeks significantly improved the skin parameters: firmness, smoothness, even skin tone/discoloration, lines/wrinkles, radiance, pore size and hydration that ranged from 51% to 78% over baseline values [9], which suggested a low dose of topical equol may benefit skin health in estrogen-deficient skin.

## 9. Future Directions

The loss of estrogen with aging, especially associated with menopause, represents a dramatic decline in skin health along with several age-related conditions, diseases, and disorders [9,23,24,25,29,32]. However, phytoestrogens in postmenopause may suggest health benefits, where the average daily dietary soy intake is between 20 and 50 mg/day in the East and Southeast Asia versus 0.1–3 mg/day in the United States and only 0.5–1 mg/day in Europe [64,154]. The relationship between diet and human health plays an important role in prevention of many age-related diseases and conditions [154,155,156,157]. Interestingly, while the recommended daily intake of isoflavones has not yet been established, the US FDA recommends an intake of 25 mg per day, which is considered to be safe [64,154]. Thus, just as the cancer incidence increases with age, the innovative strategy to combat this disease now includes combinations therapies, where estrogen receptor degraders and aromatase inhibitors represent future clinical treatments [158]. For resveratrol, oral and topical formulas combining additional active ingredients have been around for some time; however, this approach has become more popular among other polyphenolic/phytoestrogen personal care products, even though it is difficult to attribute the improvements in skin health to specific active ingredients. In this regard, analogs of resveratrol for skin lightening cosmetic applications have been reported [159]. Additionally, a recent 12 week single-center study of 42 women (40 to 70 years of age, which most were postmenopausal, 78%) that applied a topical nutricosmeceutical containing equol (at 0.15%) plus other natural active ingredients (e.g., grape seed extract, vitamin C, hyaluronic acid) showed remarkable improvement over topical equol treatment alone [in 49 women, 40–70 years of age (in the equol treatment only group)] for skin firmness, smoothness, tone/discoloration, wrinkles, radiance, pore size and hydration [160]; see Table 2. Undoubtably, this represents a synergistic mechanism by which nutricosmeceutical formulations can yield significantly greater improvement in skin health parameters compared to single active ingredient formulations. Specifically, this study comparison represents an almost 40% average increase among the eight skin parameters for overall improvement; see Table 2. This is especially true, when one considers the incorporation of marine skin molecules [4,5,161] with plant-derived compounds and other natural ingredients [162] for future innovations in personal care products including exposure to air pollution [104,163,164,165].

## 10. Conclusions

Perspective is paramount in understanding the controversies associated with superfoods, phytoestrogens, and endocrine-disruptors because they have both positive and negative connotations even when the same food source contains labels denoting each type of connotation. Resveratrol and equol are phytoestrogens. Everyone is exposed to and consumes these molecules everyday regardless of age, gender, or geographic location around the world, and how we understand their effect(s) is a matter of perspective. To understand phytoestrogens, one must understand: (a) estrogen, (b) estrogen receptors (ER), (c) characteristics of estrogen-deficient skin, (d) the properties of phytoestrogens binding to ERs, (e) how misconceptions/misinformation occur, especially about phytoestrogens and even in the safety of human food products, (f) the elucidation of the in vitro and clinical evidence for resveratrol and equol in combating skin aging, especially after menopause and finally, (g) the combination of active (mineral, plant-derived and marine) skin ingredients appears to be the innovation of the future for personal care products for oral and topical skin applications that include exposure to air pollution.

## Figures and Tables

**Table 1:** Human Skin Gene Expression Among Resveratrol, 4′ Acetoxy-Resveratrol, R-Equol, Racemic Equol and S-Equol. as a Percentage Increase (+) or Decrease (−) Compared to Controls.

Gene	Resveratrol	4′ Acetoxy-Resveratrol	R-Equol	Racemic Equol	S-Equol
Anti-Aging ▲ and Aging Factors ▼					
SIRT1 ▲	+180	+335	NA	+190;↑ with Resveratrol •	NA
PCNA ▲	+780	+540	+235	+285 to +300	+325
NGF ▲	+800	+672	+3350	+2860	+1620
5α-Reductase ▼	NSA	NSA	NA	−180	NA
S100 A8 ▼	−340	−270	−2050	−1000 to −2230	−580
S100 A9 ▼	−290	−160	−1850	−1180 to −2250	−525
Extracellular Matrix Proteins: (that enhance collagen & elastin)					
COLI alpha 1	+225	NSA	+210	+235	+185
COL III alpha 1	+230	+220	NSA	NSA	NSA
COL IV alpha 1	+160	+170	NSA	+210	NSA
Elastin	+180	+280	NSA	+175 to +270	+1
TIMP 1	+215	+250	+2	+200 to +540	+150
LOX	+180	+190	NA	NA	NA
Degrading Collagen/Elastin Enzymes					
MMP 1	−180	NA	−890	−540	−325
MMP 3	NSA	NA	−885	−800	−330
MMP 9	−485	NA	−1375	−1010 to −1080	−710
Antioxidants ■ plus Heavy Metal Binder/Anti-inflammatory Mediators ◊					
CAT ■	+180	+160	NA	NA	NA
SOD 1 ■	+160	+160	NA	+200	NA
SOD 2 ■	+160	+170	NA	+130 to +200	NA
TXNRD1 ■ ◊	NSA	NSA	NA	+215 to +250	NA
MTH 1 ■ ◊	+4100	+6400	+2100	+1800 to +2310	+3840
MTH 2 ■ ◊	+200	+340	NA	+510	NA
Inflammatory Factors					
IL-1A	−2200	−1010	−1385	+1700	−990
IL-1R2	−590	−190	−1730	+2200	−1675
IL-6	−3200	−3520	−550	−455	−375
IL-8	−790	−380	−295	−345	−445
TNFRSF1A	−160	−140	−310	−250 to −665	−205
COX 1	NSA	NSA	−360	−265	−200
COX 2	NSA	−170	NSA	+155	NSA

Data redrawn from [31,109,129,131]. • data from [130]. 4′ acetoxy-resveratrol was selected because it was the most promising among the various resveratrol analogs tested [109,121]. Since multiple experiments were run in the same laboratory under similar conditions (1% of the test compound applied and then exposed to human skin cultures for 24 h), the largest significant percent stimulation or inhibition for: (a) resveratrol is shown in red, (b) 4′ acetoxy-resveratrol shown in gold, (c) R-equol is shown in blue, (d) racemic equol shown in green, and (e) S-equol shown in gray (label), where data are available for a given gene biomarker for comparisons among the polyphenolic/phytoestrogens tested. Data shown in black for a given gene biomarker among the compounds tested indicates no significant difference for the largest percent stimulation or inhibition of the displayed quantified values; however, values may be higher or lower among all the compounds tested, but the highest or lowest rating was not color-coded due to no significant difference in the values. Remarkably, S-equol did not reveal any values that displayed a significant stimulation (highest) or significant inhibition (lowest) levels among the test compounds for any of the skin biomarkers tested. Note: racemic equol with more than one value (range) indicates the results of multiple independent experiments. NA = not assayed; NSA = no significant change or alteration in the gene tested. Gene Symbols/Function: SIRT 1 = sirtuin activator 1 or NAD-dependent protein deacetylase sirtuin-1, an anti-aging factor; PCNA = proliferating cell nuclear antigen, involved in DNA repair; NGF = nerve growth factor, involved in skin/tissue repair and neurotrophic factor; 5α-reductase 1 = the steroid enzyme that converts testosterone to the more potent androgen, 5α-dihydrotestosterone (5α-DHT) that inhibits dermal health; S100 A8 and A9 = calcium-binding proteins (A8 and A9), related to skin aging, skin inflammation and photoaging; collagen type I alpha 1, collagen type III alpha 1 and collagen type IV alpha 1, collagen dermal fibers for structural support; Elastin = elastic fibers that provide skin bounce-back (elasticity) after deformation; TIMP 1= Tissue Inhibitor of Matrix Metalloproteinase 1, an enzyme that inhibits the action of MMPs; LOX = Lysyl Oxidase, cross links collage and elastin fibers; MMP = matrix metalloproteinases (1, 3 and 9), an enzyme that breakdown collagen/elastin fibers; CAT = catalase, an antioxidant enzyme that protects against oxidative stress; SOD = superoxide dismutase (1 and 2), an antioxidant enzyme that protects against oxidative stress; TXNRD1 = thioredoxin reductase 1, an antioxidant enzyme that protects against oxidative stress; MTH = metallothionein (1 and 2), heavy metal binding protein and an anti-inflammatory mediator; IL-1A = interleukin-1 A, inflammatory factor; IL-1R2 = interleukin 1 receptor II, inflammatory factor; IL-6 = interleukin 6, inflammatory factor; IL-8 = interleukin 8, inflammatory factor; TNFRSF1A= tumor necrosis factor receptor super family 1A, inflammatory factor that can activate NF-Kβ; COX 1 or Cox 2 = cyclooxygenase 1 or 2, inflammatory enzyme.

**Table 2:** Self-Assessment Questionnaire Analysis—Facial Features [160] Efficacy: percentage of subjects that perceived improvement with the equol plus natural ingredients (ENI) over the baseline (parameters 1–8 data not shown) and/or compared to the equol treatment alone (EA) at 12 weeks (see below).

Week 12	Equol Plus Natural Ingredients (ENI)	Equol Alone	Increase Over (EA)
1. Skin Firmness	91% *	73%	18%
2. Smoothness	98% *	63%	35%
3. Even Skin Tone	98% *	57%	41%
4. Frown Lines/Wrinkles	89% *	65%	24%
5. Radiance/Brightness	98% *	63%	35%
6. Pore Size	93% *	20%	73%
7. Spots/Discoloration	84% *	31%	53%
8. Hydration	95% *	61%	34%
Number of Subjects	42	49	
Mean Age (years + SEM)	57.3 + 7.3	56.7 + 8.78	
Age Range (years)	40–70	40–70	
Caucasian (number subjects)	23	30	
Chinese (number subjects)	4	8	
Japanese (number subjects)	15	11	

Amenorrheic for at least 2 years in the ENI group was 78% versus the EA group that was 77%; *= significantly greater compared to equol treatment alone (EA).

## Data Availability

The data presented in this study are available on request from the corresponding author. Some data may not be publicly available due to funding sponsor intellectual property agreement.
